# The Politics of Transnational Advocacy Against Chinese, Indian, and Brazilian Extractive Projects in the Global South

**DOI:** 10.1177/10704965211019083

**Published:** 2021-05-15

**Authors:** Leah Shipton, Peter Dauvergne

**Affiliations:** 1 Department of Political Science, 8166 University of British Columbia, Vancouver, Canada

**Keywords:** transnational advocacy networks, extraction, Brazil, Russia, India, China, and South Africa, Ecuador, Ethiopia, Mozambique

## Abstract

Activists in the global South have been navigating two powerful trends since the mid-1990s: intensifying state repression and rising investment in extractive projects from the emerging economies of Brazil, Russia, India, China, and South Africa (BRICS). In this context, this article explores the underlying forces determining the formation, endurance, and power of BRICS–South transnational advocacy networks (TANs) opposed to BRICS-based corporate extraction in the global South. By analyzing activism against Chinese, Indian, and Brazilian extractive projects in Ecuador, Ethiopia, and Mozambique, respectively, the research reveals the critical importance of domestic politics and civil society characteristics in both the BRICS and host states for shaping BRICS–South TANs, including which groups assume leadership, the extent of cross-national cooperation, and the role of nonprofits headquartered in the global North. The findings uncover core reasons for the variable resiliency and capacity of BRICS–South TANs, opening up new avenues of research and offering valuable insights for activists and policymakers.

Intensifying suppression of dissent and the growing power of emerging economies over the past few decades have significantly altered the conditions and strategies of activists campaigning against transnational corporate mining, plantations, and land grabbing in the global South. Since the mid-1990s, and especially since the terrorist attacks of September 11, 2001, governments—from mature democracies to authoritarian regimes—have sought to constrain anti-government protests and nongovernmental organizations (NGOs) (Dupuy et al., 2016; Howell, 2012; Kreienkamp, 2017). Dupuy et al. (2015, p. 420), for instance, found that NGOs across a wide range of countries have been subject to a “regulatory offensive,” with governments harassing activists and constricting the financial, administrative, and communicative capacities of NGOs. By criminalizing advocacy, cutting off foreign funding, increasing surveillance, and imposing arduous reporting rules, this regulatory offensive has undermined the capacity of local NGOs to forge coalitions and mobilize support, both domestically and internationally (Christensen & Weinstein, 2013; Dupuy et al., 2016; Matejova et al., 2018). Activists trying to protect land in resource-based economies in the global South are facing especially grave risks. The large number of murdered environmental defenders (212 reported in 2019) is but one indicator (Global Witness, 2020).

Over this time, too, foreign investment and development cooperation for extractive projects between Brazil, Russia, India, China, and South Africa (BRICS) and non-BRICS states of the global South (what we call “South states”) have been steadily rising (Inoue & Vaz, 2012; Lauria & Fumagalli, 2019; Milhorance & Bursztyn, 2017; Nayyar, 2016).^1^ BRICS–South agreements to facilitate extractive projects in South states have proliferated too, often under conditions that disadvantage the latter (e.g., tax exemptions and low royalties) and raise alarms of subimperialism and neocolonialism (Gray & Gills, 2016).^2^ When noting this trend, we are defining extractive projects broadly, including mining, logging, fracking, oil drilling, water diversion, and agricultural plantations. Also, we are using the acronym BRICS, not to refer to a quasi-organization or informal negotiating structure, but rather as shorthand for the emerging economies of Brazil, Russia, India, China, and South Africa, “a group of regional leaders […] each of which has the sum of the parameters of an economic, political, military and civilizational center of power in their regions” (Lagutina, 2019, p. 445).^3^

The growth in BRICS extractive projects in South states has created a need for stronger BRICS–South transnational advocacy networks (commonly referred to as TANs, which we define in full in the next section). Yet, at the same time, activists in both emerging and developing economies are campaigning in increasingly repressive political settings and facing grave dangers from state security forces and private corporations, especially those opposing large-scale extractive projects (Freedom House, 2021; Global Witness, 2020).^4^ Caught at the intersection of these trends, how are NGOs and activists in BRICS and South states navigating their respective repressive settings to mobilize, separately or jointly, against BRICS-financed and -managed extractive projects in the global South? What role do NGOs headquartered in the global North assume in burgeoning BRICS–South alliances? Employing case studies of activism against Chinese, Indian, and Brazilian extractive projects in Ecuador, Ethiopia, and Mozambique, respectively, this article explores these questions.

The mobilization patterns of BRICS–South TANs, as this article shows, reflect the conditions and characteristics of civil society in both the BRICS and host state. In saying this, we are not assuming civil society is fully autonomous from the state and market. Nor are we associating civil society exclusively with democracy and individual rights as this can exclude influential forms of associational life in the global South, such as traditional authorities (e.g., chiefs and sharia courts), religious groups, ethnic-based associations, and community self-help groups (Daniel & Neubert, 2019). To capture the diversity of the global South, we see civil society and TANs as potentially comprising “a range of entities of different types, sizes, purposes, and levels of formality, including community or grassroots associations, social movements, labor unions, professional groups, advocacy and development NGOs, formally registered nonprofits, social enterprises, and many others” (Edwards, 2011, pp. 7–8).

Four major factors, we argue, influence how ready, willing, and able civil society in the global South is to pursue BRICS–South transnational activism: the extent of legal protections and political repression within each state, the degree of domestic political and economic stability, the experience of NGOs and Indigenous groups in resisting extractive projects at home and abroad, and the priority that local NGOs and Indigenous peoples place on building South–South solidarity. When conditions are favorable in the South state, but not the BRICS state, activism tends to be led by and take place in the South state (as in the case of China–Ecuador). In the reverse scenario, a substantial amount of the advocacy tends to take place in the BRICS or internationally (as in the case of India–Ethiopia). When conditions are reasonably favorable in both countries, civil society organizations tend to deploy strategies independently and jointly in the BRICS and South context, with support from international and North-based NGOs (as in the case of Brazil–Mozambique).

Drawing on scholarship, reports, and other documentation, this article adds another layer of understanding to the literature analyzing BRICS–South TANs (e.g., Cezne, 2019; Hochstetler, 2002; Moreira et al., 2019; and Rodrigues, 2004). This includes insights into the political conditions and civil society characteristics that facilitate or impede the participation of both BRICS and South-based civil society organizations in these TANs. Accompanying this main contribution is an illustration of how North-based civil society organizations and international NGOs can support the development and maintenance of BRICS–South TANs by facilitating connections, funding meetings and field visits, and raising environmental awareness, among other activities.

We begin our analysis with a brief review of the literature on South–South TANs, noting the relative lack of research on BRICS–South TANs. Next, to add new layers of understanding of the characteristics and influence of BRICS–South TANs, we investigate advocacy networks opposing BRICS extractive projects across three cases: China–Ecuador, India–Ethiopia, and Brazil–Mozambique. We conclude by comparing our case study findings and reflecting on their implications.

## BRICS/South–South TANs

Transnational advocacy networks comprise “actors working internationally on an issue, who are bound together by shared values, a common discourse and dense exchanges of information and services” (Keck & Sikkink, 1999, p. 89). This article focuses on TANs that form in response to, and with the aim of addressing, the environmental and social consequences of extractive projects in the global South. A common strategy of such TANs is known as the “boomerang strategy,” which occurs when local actors facing state repression seek support from (i.e., throw a boomerang to) international allies who return that support (i.e., throw the boomerang back) by pressuring key state actors, corporations, or intergovernmental bodies (Keck & Sikkink, 1998; McAteer & Pulver, 2009; Park, 2005). Activists in the global South have tended to use the boomerang strategy to seek support from NGOs headquartered in the global North (den Hond & de Bakker, 2012; Keck & Sikkink, 1998; McAteer & Pulver, 2009; Risse et al., 1999). In recent years, however, South-based activists opposing BRICS extractive projects have been increasingly throwing boomerangs to BRICS-based NGOs.

Throwing boomerangs to seek overseas allies can be highly effective although, as many scholars warn, vigilance is necessary to avoid overemphasizing the influence of transnational alliances and underestimating the importance of domestic activism and political conditions (Hochstetler, 2002; Minami, 2019; Rodrigues, 2004, 2015; Rosenberg, 2018; Temper, 2019). Additionally, we need to keep in mind that the boomerang can be inversed: meaning that a North-based or BRICS-based NGO can also initiate contact with South-based actors to pursue their own political goals at home (Pallas, 2017; Temper, 2019).

Politics within TANs profoundly shape mobilization patterns, too. Founding members of TANs or its more powerful members (e.g., international NGOs), for instance, can exercise disproportionate influence on a network’s agenda and focus (Carpenter, 2010; Lake & Wong, 2009). This is true, too, for South–South advocacy networks, which Moreira et al. (2019, p. 80) define as “subcategories of TANs whose information, symbolic, leverage, and accountability politics derive primarily from the cross-border activities and interactions of Southern actors, including grassroots social movements, research and advocacy organizations, and local and national NGOs.”

The scholarship on TANs reveals unique challenges to the formation and maintenance of BRICS/South–South TANs. The tendency for South investments to flow through national development banks (NDBs), rather than multilateral development banks, limits the access points for these advocacy networks (Moreira et al., 2019). In the case of the Brazilian National Development Bank, for instance, Sierra and Hochstetler (2017) find that indirect strategies to raise public awareness and put an issue (e.g., transparency and environmental standards) on the judicial or legislative agenda have been more effective than directly targeting the bank. Also problematic for BRICS–South TANs is the lack of transparency in BRICS overseas expenditures and BRICS financial institutions (John, 2012; Mawdsley & Roychoudhury, 2016; Rodrigues, 2016).

In response, BRICS-based civil society actors have organized counter-summits to BRICS and NDB forums (Bond & Garcia, 2020). Still, public pressure in the global South for ethical foreign policy and transnational solidarity is only just emerging (Mawdsley & Roychoudhury, 2016; Rodrigues, 2016). Patriotism and economic interests, especially among the elite, may also be further stifling public concern over the consequences of overseas investments (John, 2012; Mawdsley & Roychoudhury, 2016). Additionally, as large-scale South–South investment is relatively new, civil society actors in South states tend to have limited financial, human, and institutional capacity to expose the consequences of this investment (John, 2012; Mawdsley & Roychoudhury, 2016; Rodrigues, 2016).

Adapting to these challenges, BRICS–South TANs have found some success. The Amazonian Hydroelectric Collective anti-dam campaign, for example, successfully influenced the Peruvian state and Brazilian corporations to cancel a series of hydroelectric projects planned in Peru (Moreira et al., 2019). Financed by the Brazilian Development Bank, the projects were established through a bilateral treaty between Peru and Brazil that largely excluded civil society participation. To oppose this project, a Brazil–Peru TAN was formed. TAN members undertook a variety of strategies—such as meeting with Peruvian elected officials, framing Brazil’s conduct as neocolonial, shaming Brazilian companies, and lobbying officials of the Brazilian Development Bank—that eventually led the Peruvian government to reject the treaty and several Brazilian companies to withdraw.

Scholarship on BRICS–South TANs, as this short overview suggests, is only just starting to emerge. Significant gaps remain in the scholarly knowledge of the formation, durability, extent of cross-national cooperation, role of North-based NGOs, and overall influence of these advocacy networks. The rest of this article builds on this early scholarship to deepen the understanding of the underlying forces shaping BRICS–South TANs campaigning against BRICS-based extractive projects in the global South. We begin with a look at campaigns to oppose Chinese extractive projects in Ecuador. As we will see next, Ecuadorian civil society organizations are leading resistance efforts with some support from NGOs in Latin America and the global North. Meanwhile, a China–Ecuador advocacy network has not formed and civil society ties between China and Ecuador remain weak.

## China–Ecuador

### Chinese Extractive Investment and Companies in Ecuador

President Rafael Correa’s decision to nationalize Ecuador’s natural resources following his 2007 election and to default on Ecuador’s foreign debt in 2008 led several oil companies to exit the country, putting Correa in need of revenue to fund his commitments to social investments and maintain his legitimacy (Gonzalez-Vicente, 2017; Nathanson, 2017b). China stepped in with energy, infrastructure, and transportation investments totaling more than US$ 17 billion between 2007 and 2017 (Nathanson, 2017a). By 2017, Ecuador’s debt to China equaled almost 40% of its gross domestic product, and Chinese companies managed and financed eight hydroelectric dams, oil concessions in Yasuni National Park, and three mining sites in the country (Koening, 2017; Nathanson, 2017a). The Ecuadorian government has facilitated Chinese investments by permitting Chinese companies to bypass environmental and social impact assessments and by deploying the police to quell community resistance (Nathanson, 2017b).

This influx of Chinese investments and companies has threatened communities, and civil society resistance has been fierce, particularly against large-scale mining projects (e.g., Mirador, Panantza-San Carlos, and Río Blanco). To make way for the projects, the Ecuadorian government and private security have forcibly displaced and burned down villages (Nathanson, 2017a). Indicative of the extent of violence, the Indigenous environmental activist José Tendetza was murdered in 2014, days ahead of his scheduled condemnation of the Chinese Mirador mining project at the Rights of Nature Tribunal in Peru (part of United Nations climate talks).

### Ecuadorian Civil Society and State Repression

Ecuadorian civil society, and in particular Indigenous groups, has a long history of activism against state threats to Indigenous autonomy (see, for example, the Confederation of Indigenous Nationalities of Ecuador) and extractive projects (see, for example, the decades long campaign against Texaco/Chevron). Thus, Ecuador’s civil society, albeit not without fractures (Widener, 2009), is relatively well equipped to mobilize against extractive projects (Widener, 2011). Ecuador’s constitution also offers some legal protections, such as the right of Indigenous peoples to free, prior, and informed consent (FPIC) and rights of nature (Republic of Ecuador, 2008).

Still, Ecuadorian civil society is navigating a context of increasing state repression. Presidents and government officials commonly seek to weaken civil society by framing environmental organizations and activists as vectors of foreign influence (Matejova et al., 2018; Widener, 2009). The Ecuadorian government has also tried to shut down and control dissenting civil society organizations. For example, in 2009, the Ecuadorian government dissolved the environmental organization, Acción Ecológica (and then reversed its decision after substantial international campaigning) (Riofrancos, 2015). Acción Ecológica was targeted again in 2016 after the Chinese mining company Explorocobres complained to the Ecuadorian government about the organization’s support for protests against their mine (Soutar, 2016). Similarly, in 2013, the government shut down the offices of the nonprofit organization Fundación Pachamama because it supported anti-oil protests by Indigenous Kichwa and Sarayaku communities (Liévano, 2019; Riofrancos, 2015).

While these assaults occurred under the tenure of President Correa (2007–2017), the trend has continued under President Lenín Moreno. In 2019, the Moreno government imposed a 2-month state of emergency to suppress protests following the adoption of austerity measures and a pro-extractivism agenda. In 2020, the government restricted the right to assemble, further threatening those resisting foreign mining on Indigenous land (International Center for Not-for-Profit Law, 2020b). The Moreno administration has also failed to implement national policies to protect Amazonian Indigenous women who are under attack for trying to defend their lands.

### Targeting China with the Boomerang Strategy

The growing role of China in extractivism has posed difficult challenges for Ecuadorian activists. Chinese firms, lenders, and policy advisors tend to form close ties with the central government and largely avoid engaging with communities (Gonzalez-Vicente, 2017; Widener, 2011). State–company alliances are certainly present in some extractive projects run by multinational corporations headquartered in the global North. To retain its principle of noninterference and avoid local politics, however, Chinese governmental and corporate actors tend to prioritize building state alliances over community relations (Zheng, 2016; Zou & Jones, 2020). This subsequently limits opportunities for local stakeholders to influence extractive projects (Gonzalez-Vicente, 2017).

At the same time, China remains largely inaccessible to the boomerang strategy. The reason is straightforward. Although Chinese civil society organizations are exerting some influence over environmental management inside China (Chen, 2010; Xie, 2011; Zhang, 2018), China does not have NGOs campaigning to improve the overseas human rights and environmental conduct of Chinese corporations (Howell, 2012; Widener, 2011; Yeophantong, 2013). In part, this is because civil society in China faces intense state repression, including government monitoring, limits on critical speech, and strict reporting rules for international collaborations and visits (Gonzalez-Vicente, 2017; Tang & Zhan, 2008). Nationalism, public disinterest, and a civil society already overwhelmed by domestic problems may also partly explain the paucity of outward-oriented civil society organizations in China (Shirk, 2007; Tao, 2008).

Adding to the challenge for outward-facing Chinese NGOs, China’s legal regulations for corporations do not extend to overseas conduct (Koop & Soutar, 2018).^5^ While the Chinese government has released guidelines for overseas conduct of its companies, they are entirely voluntary and largely unenforced (Koop & Soutar, 2018). Unlike in the global North, where civil society occasionally manages to hold extractive companies accountable to voluntary guidelines, there is not a network of Chinese civil society organizations able to do the same.

In short, China’s state-alliance approach, lack of regulatory controls on overseas corporate conduct, and the limited presence of outward-oriented Chinese activism make it a hard-to-hit target for the boomerang strategy. Consequently, Ecuadorian civil society has been left to defend against the encroachment of Chinese extractive projects with little direct access to Chinese companies, state officials, or civil society actors.

### Strategies of Resistance

The strategies used by Ecuadorian civil society to resist Chinese extractive projects have been primarily based in the domestic sphere. This reflects the strength of Ecuadorian civil society and availability of domestic legal protections on the one hand, and the inaccessibility of Chinese civil society alliances and legal recourse, on the other. The tactics of Ecuadorian activists are well-coordinated and manifold. They have targeted Chinese extractive sites and organized large-scale demonstrations (such as the 2012 March for Water, Life, and the Dignity of Peoples by the Confederation of Indigenous Nationalities of Ecuador). They have showcased biodiversity loss and reported on the socioenvironmental impacts of Chinese extractive projects (Gonzalez-Vicente, 2017; Riofrancos, 2015). And they have organized tribunals to help local people express their concerns about Chinese mining projects (Nathanson, 2017a, 2017b).

Leveraging the 2008 constitutional right to FPIC, Indigenous communities have also gone to court to claim the government violated the rights of nature as well as failed to protect their constitutional right to FPIC before awarding mining concessions to Chinese firms (Kauffman & Martin, 2017; Liévano, 2019). The courts have ruled in favor of the Kichwa and Cofán Indigenous peoples, agreeing they were inadequately consulted before the awarding of mining concessions in their territories. The Waoroni Indigenous group also won a lawsuit in 2019 against the Ecuadorian government for failing to obtain their FPIC before opening their territory to oil exploration. Not all lawsuits have been successful. The people of Cascomi, for instance, sued the Ecuadorian government following forced evictions associated with the Chinese-financed and -operated Mirador mining complex (Gonzalez-Vicente, 2017; Liévano, 2019). The court concluded the Cascomi were not Indigenous, and therefore ruled the evictions were lawful.

In addition to these domestic approaches, Ecuadorian NGOs have aligned with other Latin American NGOs to raise international awareness. This includes documenting the human rights violations of Chinese extractive projects for consideration for China’s Universal Periodic Review of the United Nations Human Rights Council (Koop & Soutar, 2018). Nongovernmental organizations from the global North have also raised awareness of Chinese corporate conduct as well as helped connect Chinese and Ecuadorian lawyers, conservationists, and journalists.

Of course, lobbying and court filings in the global North do not necessarily mean North-based firms will perform any better when mining, logging, or drilling for oil in the global South. Showcasing this activism in the global North is only meant to highlight how Ecuadorian environmental defenders lose this avenue of resistance with China’s entry into overseas extraction. In the case of Chinese-based companies, as this section shows, Ecuadorian civil society has been unable to access Chinese courts or government agencies. Nor has Chinese civil society, whether for lack of interest or capacity, widely campaigned in support of Ecuadorian campaigns against Chinese extractive projects.

Instead, Ecuadorian civil society has done the “heavy lifting” in resisting Chinese-based firms in Ecuador with modest backing from Latin American and North-based NGOs. In contrast, as we’ll see next, Ethiopian civil society has struggled to sustain campaigns against Indian-based extractive projects in Ethiopia, relying primarily on TANs to pressure firms, and routinely tossing boomerangs to seek support from international, North-based, and Indian-based civil society organizations.

## India–Ethiopia

### Indian Extractive Investment and Companies in Ethiopia

Between the mid-1990s and 2011, and especially from 2008 to 2011, the Ethiopian government leased an estimated 3–3.5 million hectares of land to foreign investors based mainly in emerging and developing economies (Anywaa Survival Organisation, 2018; Rahmato, 2014). This land has been used to cultivate food and grow industrial and biofuel crops. India has been a major investor, motivated by domestic food insecurity and water scarcity as well as a high rate of return (Cheru, 2016; Viswanathan & Mishra, 2020). This includes, for example, a line of credit from the Export-Import Bank of India to develop Ethiopia’s sugar industry (Beri, 2016; Export-Import Bank of India, 2016).

Two Indian companies, Overseas Infrastructure Alliance and Uttam Sucrotech, won contracts for projects funded by this line of credit, illustrating the importance of the Export-Import Bank in helping Indian companies access Ethiopia (Cheru, 2016). At the time of writing, the Land Matrix database reports 17 land deals with 14 Indian companies (including one private bank), covering 176,000 ha of land for food and nonfood agricultural exports (The Land Matrix, 2021). For their part, the Ethiopian government has attracted foreign investors by offering large tracts of land, tax exemptions, and a contractual commitment to keep locals off the leased land (thus, justifying the government’s forced eviction of Indigenous groups and suppression of any resistance) (Rowden, 2011).

### Ethiopian Civil Society and State Repression

Ethiopian civil society flourished from the mid-1990s to mid-2000s, illustrated in part by the expansion from 70 local NGOs in 1994 to 2,275 in 2009 (Dupuy et al., 2015). Conditions worsened, however, after the Ethiopian government blamed NGOs for election violence in 2005. Following this election, the government implemented the 2008 Mass Media Proclamation, 2009 Anti-Terror Proclamation, and the 2009 Charities and Societies Proclamation, all of which restricted the work of rights-oriented journalists and NGOs. This included limiting access to foreign funding, increasing surveillance, and torturing and jailing activists, to the point of driving many into exile (Gebre, 2016; Ibreck & de Waal, 2015; Mittal, 2018).

By 2011, more than 500 Ethiopian NGOs had shut down in response to this crackdown (Dupuy et al., 2015). Many of those remaining “rebranded” as service providers or focused on less controversial issues (Dupuy et al., 2015; Gebre, 2016). The few NGOs that continued to work on contentious topics had difficulty generating local support and funding, and their international allies were unable to apply significant pressure on the Ethiopian government, in part because of the suppression of NGOs inside Ethiopia (Dupuy et al., 2015).

These repressive laws have facilitated land grabbing of Indigenous territory, particularly impacting Indigenous people (e.g., Anuak, Mursi, and Bodi) of the Gambella region and Lower Omo Valley. The villagization program has been one of the government’s instruments to enact this violence. This program has forcibly moved Indigenous peoples living on so-called “underutilized” land into villages, often with substandard medical, educational, and other services. Without consent, the government has then leased their land for plantations (International Work Group for Indigenous Affairs, 2020b; Mittal, 2018; Rahmato, 2014).

These land grabs have adversely impacted between 200,000 and 500,000 people (Rahmato, 2014). Describing it as economic development, the Ethiopian government has used villagization and agricultural investments to “thicken” the state’s presence—and subsequent capacity to repress—in the country’s hinterlands (Regassa et al., 2019). Adding to the government’s repressive capacity, Ethiopia does not have legislation protecting Indigenous people and has not ratified the Indigenous and Tribal Peoples Convention (ILO Convention 169), leaving gaps in legal protections for these groups (International Work Group for Indigenous Affairs, 2020a).

Prime Minister Abiy Ahmed’s appointment in 2018 eased the constraints on civil society somewhat, although repressive laws and state violence targeting activists and Indigenous groups opposing state-backed extractive projects endure (Freedom House, 2021). For example, in 2019, as interest in land of the Lower Omo Valley for sugarcane plantations increased, the Ethiopian government disarmed Bodi, Mursi, and Suri Indigenous groups to weaken their ability to defend their ancestral territory (International Work Group for Indigenous Affairs, 2020b). A turn to authoritarian tactics and the Tigray War have been further constraining the activities of local NGOs over the past few years (Freedom House, 2021).

Overall, the relatively weak position of Ethiopian rights-oriented civil society and limited legal protections for Indigenous peoples put activists campaigning against land grabbing in the precarious position of facing human rights abuses with few options for recourse.

### Targeting India with the Boomerang Strategy

Compared to China, India is a more accessible target for the boomerang strategy. Nonetheless, in recent years, Indian civil society has been under increasing restraints as the Indian government favors service NGOs and frames rights-based NGOs as disloyal and “antinational,” especially those with foreign ties (Matejova et al., 2018; Mawdsley & Roychoudhury, 2016). To host international meetings, moreover, Indian NGOs must obtain approval from several government entities (e.g., Ministries of Home and External Affairs). The Indian government, too, has been impeding efforts by Indian NGOs to connect with peoples displaced by Indian foreign investment and development projects, including Indigenous groups in Ethiopia (Mawdsley & Roychoudhury, 2016). India’s lack of robust laws to regulate overseas corporate conduct further reduces the capacity of Indian allies of Ethiopian civil society to challenge the legality of Indian extractive projects in Ethiopia (Deva, 2016).

Despite these barriers, Indian civil society is in the early stages of developing an activist network that targets Indian corporations overseas (Mawdsley & Roychoudhury, 2016; Rowden, 2011). Aiding the turn toward transnational advocacy in this area is Indian civil society’s experience organizing against domestic land grabs by foreign corporations. To this point, during a 2011 New Delhi protest against the Indian government’s proposed Land Acquisition Act, civil society organizations (e.g., National Alliance of People’s Movement, Indian Social Action Forum, and Kalpvriksha) and independent activists spoke against land grabbing by Indian companies in Africa as well as India’s development policies enabling this conduct. These activists spoke of a common struggle of Indian and African civil societies.

In 2013, the US-based Oakland Institute facilitated a meeting in India between these Indian activists and Indigenous Ethiopians impacted by Indian land grabbing in Ethiopia (Mawdsley & Roychoudhury, 2016). Following this event, three Indian NGOs published a briefing note outlining the human rights and environmental abuses arising from Indian agricultural investments in Ethiopia (Kalpavriksh et al., 2018). This example illustrates that even if companies or governments in the global North are not active in a particular extractive project, North-based NGOs may play a role in developing BRICS–South TANs.

To try to shape India’s overseas development policies, other Indian civil society organizations have participated in initiatives such as the Forum on Indian Development Cooperation (Mawdsley & Roychoudhury, 2016). In another example, the international NGO Saferworld and the Indian think tank Observer Research Foundation brought together a panel of experts and government officials to discuss Indian development cooperation in conflict-affected states (Sharan et al., 2013). Reflecting on the experience of Chinese firms managing protests against extractive projects in Myanmar, South Sudan, and Sudan, the group discussed how India’s development principle of noninterference may face challenges in these contexts and that consulting with communities (rather than solely with central authorities) and minimizing community displacement from land are important for avoiding conflict.

In short, while Indian civil society faces barriers to politically organizing and shaping India’s development policies, the space permitted to organize and the experience in resisting domestic land grabs give them considerable potential to develop advocacy networks with civil society organizations in countries affected by Indian extractive companies and investment.

### Strategies of Resistance

On the other hand, state repression of NGOs and Indigenous groups in Ethiopia severely limits the domestic strategies available to resist Indian investments (Schapper et al., 2020). Aside from protesting and vocalizing discontent, affected people have used, in the words of James Scott (1998), “everyday forms of resistance,” such as stealing or damaging the property of investors, grazing cattle on industrial plantations, and strategically deploying development language at public meetings to mollify state officials (Rahmato, 2014; Regassa et al., 2019).

Foreign NGOs have been crucial for filling the advocacy vacuum created by Ethiopia’s repressive setting. Besides organizing meetings between Indian organizations and Indigenous Ethiopian activists, international NGOs such as the Oakland Institute and GRAIN have helped raise awareness by researching the human rights violations associated with extractive projects in Ethiopia (Rowden, 2011). North-based Ethiopian diaspora organizations, such as the Solidarity Movement for a New Ethiopia and Anywaa Survival Organisation, share community testimonies—for instance, Obang Metho of the Solidarity Movement for a New Ethiopia spoke to the Africa Congress of his Anuak community’s experience—to inform Indian civil society and pressure governments in the global North to address the crisis in Ethiopia (Anywaa Survival Organisation, 2018; Mittal, 2018; Rowden, 2011).

North-based academics, such as Concerned Scholars for Ethiopia, have also spoken out against the Ethiopian government in solidarity with Indigenous communities impacted by state violence associated with extractive projects (International Work Group for Indigenous Affairs, 2020b). While these organizations have tended to target the Indian and Ethiopian governments, they have come together on several occasions to single out the Indian company, Karuturi Global, for its land acquisitions (Anywaa Survival Organisation, 2019; Chandran & Gardner, 2017; Tax Justice Network et al., 2014). Some of this international scrutiny has pushed the Ethiopian government to monitor the environmental impacts of agricultural investments more closely and in the case of the Karuturi Global, forced it to return some land (Cheru, 2016).^6^

Civil society organizations from the global North and India, then, are in relatively prominent positions in the TANs opposing Indian extractive projects in Ethiopia as Ethiopian activists struggle to survive within a highly repressive society. The nature of TANs opposing Brazilian extractive projects in Mozambique, as the next section discusses, is different again. Here, backed by an array of North-based NGOs, civil society organizations from both countries have been participating actively in these networks, although rising state repression and political crises in both countries have been weakening the power of these advocacy networks in recent years.

## Brazil–Mozambique

### Brazilian Extractive Investment and Companies in Mozambique

Brazil’s participation in South–South development increased significantly during President Luiz Inácio “Lula” da Silva’s tenure from the beginning of 2003 to the end of 2010 (Dauvergne & Farias, 2012). The government extended diplomatic ties in Africa to strengthen its influence as an emerging power and increase its export of goods and services (Farias, 2018). Mozambique has been one of Brazil’s largest recipients of foreign assistance, including technical cooperation, loans, and investment (Schlesinger, 2014; Wolford & Nehring, 2015). In 2009, Brazil became Mozambique’s leading source of foreign direct investment (FDI), and by 2011, Brazil’s development agency had allocated approximately US$ 32 million to Mozambique (Durán & Chichava, 2017; Nogueira et al., 2017; Schlesinger, 2014) (foreign assistance to Mozambique, however, did decline in 2014 as Brazil attended to its own political and economic crises) (Nogueira et al., 2017).

Two extractive projects are particularly notable in Brazilian–Mozambican relations: the Vale mining project and the ProSAVANA agricultural project. In 2004, with diplomatic assistance from the Brazilian government, the Brazilian mining giant Vale won a bid for the Mozambican Moatize coal reserves (Cezne, 2019). Operations commenced in 2011, marking one of the largest FDIs in Mozambique’s history (Cezne, 2019). Vale’s coal mining, and associated infrastructure projects (e.g., the Nacala Logistics Corridor), has been widely criticized for displacing communities and polluting surrounding ecosystems (Cezne, 2019; Lesutis, 2019a, 2019b).

The ProSAVANA agricultural project, a joint collaboration between the Brazilian, Japanese, and Mozambican governments and private sector, commenced in 2009. The project was modeled after the Japanese–Brazilian PROCEDER project, a commodity export-oriented agribusiness program implemented in Brazil’s Cerrado region during the 1970s. Like Vale’s activities and the PROCEDER project, ProSAVANA forcibly displaced communities, increasing food insecurity, among other social ills (Aguiar & Pacheco, 2016; Cezne, 2019; Schlesinger, 2014).

### Mozambican Civil Society and State Repression

NGOs in Mozambique began to expand following the 1992 Peace Accords ending the Mozambican civil war, but they still navigate a repressive political setting. The government has a history of only supporting nonthreatening NGOs, such as service providers, and legitimizing decisions with superficial participatory processes (Lorch & Bunk, 2017). Organizations critical of the government, meanwhile, have been thwarted, harassed, and accused of being under foreign influence (USAID & FHI360, 2018).

Consequently, civil society organizations in Mozambique tend to align themselves with state policy or adapt to the political constraints and, only rarely, openly resist the government (Lorch & Bunk, 2017). High profile assaults and assassinations of human rights and environmental defenders in 2018 and 2019 have raised concerns that space for rights-oriented activists is shrinking even further. Additionally, a reduction in foreign and government funding is threatening the viability of many civil society organizations (USAID & FHI360, 2018).

Resisting large-scale extractive projects in Mozambique has become especially dangerous since foreign investment began booming a decade ago (Cezne, 2019; Frontline Defenders, 2020). Yet, at the same time, human rights abuses associated with these projects have mobilized Mozambican civil society to form domestic and international coalitions in ways previously unseen in the country (Cezne, 2019; Milhorance & Bursztyn, 2017). Mobilization rose in tandem with the increased presence of Brazilian investment and firms in the region, with Vale’s coal mines and the ProSAVANA agricultural project acting as key catalysts (Frontline Defenders, 2020).

### Targeting Brazil with the Boomerang Strategy

Brazil is a largely accessible boomerang target. For one, Brazil has a vibrant civil society with a long history of mobilizing against extractive projects within the country and alongside the civil societies in Latin America more broadly (Cezne, 2019; Moreira et al., 2019; Rodrigues, 2004). Thus, in addition to having a strong domestic coalition for anti-extractive organizing, Brazil’s civil society has also been outward-facing, even if primarily toward Latin American countries, as well as receptive to forming transnational linkages with South-based civil society organizations mobilizing against Brazilian extractive projects.

Although open to participating in South–South TANs, Brazil’s civil society has been under duress in recent years. The 2002 election of President Lula of the Partido dos Trabalhadores (Workers’ Party) brought cautious hope of greater citizen participation in decision making and a reduction of inequities (Dagnino & Teixeira, 2014; Klein, 2015). The Lula administration, however, pursued an aggressive development strategy, which continued under President Dilma Rousseff (2011–2016). This strategy aimed to accelerate economic growth with state intervention in concert with the private sector (including privatization of state enterprises) (Hochstetler & Montero, 2013; Klein, 2015). Under the Growth Association Program, large energy infrastructure projects, such as the controversial Belo Monte dam, were a key feature of this development strategy (Bratman, 2014; Klein, 2015).

The Lula administration did make strides in formalizing civil society participation in public policy discussions at the federal level. The extractive-based development strategy, however, has created tension with (and among) segments of Brazilian civil society concerned with protecting Indigenous peoples and the environment. That tension played out, for example, when the Brazilian government ignored the concerns of some civil society organizations regarding the social and environmental impacts of the Belo Monte dam (Dagnino & Teixeira, 2014).

President Jair Bolsonaro, who came to power in 2019, has been openly hostile to civil society organizations, especially environmental NGOs (International Center for Not-for-Profit Law, 2020a). At the same time, the number of foreign- and domestic-funded extractive projects has been rising and environmental and social protections have been falling (Cezne, 2019). The COVID-19 pandemic of 2020–21 has added to the logistical and financial challenges for NGOs in Brazil. Understandably, Brazilian NGOs in recent years have been struggling to fight domestic and extraterritorial extractive battles in tandem.

Critics argue that Brazil’s framework for development cooperation requires stronger regulatory guidelines, greater transparency, and further clarity on the distribution of responsibilities (Aguiar & Pacheco, 2016; Cabral & Leite, 2015; Schlesinger, 2014). These failings in part reflect the absence of vigorous public debates in Brazil on how to approach development cooperation as well as the Brazilian public’s relatively low interest in foreign policy issues (Cabral & Leite, 2015; Schlesinger, 2014). Despite this, Brazilian civil society, whether motivated by justice-oriented international solidarity or the consequences of overseas spending on domestic capacity, has increasingly called for greater accountability and transparency in Brazil’s foreign policy, including development-related activities (Cabral & Leite, 2015). Some progress has been made. In 2018, for instance, the Brazilian government introduced a decree (no. 9571) that outlines voluntary guidelines for companies to protect human rights within Brazil and reaffirms state obligations to protect human rights in the context of business activities (Denny, 2019). This decree, however, does not extend to the conduct of Brazilian companies overseas.

In brief, although Brazil lacks extraterritorial legal obligations and its civil society is navigating a context of increasing state repression and resource extraction, the enthusiasm of NGOs to build transnational linkages and their experience resisting domestic extractive projects make the country a relatively accessible target for building BRICS–South TANs.

### Strategies of Resistance to Brazilian Extractive Investment and Companies

For Brazilian NGOs attentive to international solidarity and shared struggle of marginalized peoples, Brazil’s foreign investment in Mozambique created an opportunity to mobilize transnationally with Mozambican and North-based civil societies (Aguiar & Pacheco, 2016; Cezne, 2019; Milhorance & Bursztyn, 2017). They did so against Vale and the ProSAVANA project from the late 2000s to mid-2010s.

Prior to Vale’s expansion into Mozambique, Brazilian civil society had long resisted the company’s domestic extractive activities. In 2009, for instance, protests against Vale’s mining operation in Canada prompted the formation of the International Articulation of those Affected by Vale (AV), which mapped out Vale’s global activities and brought together activists and organizations concerned with Vale’s conduct (Cezne, 2019). Through this network, Brazilian civil society became aware of Vale’s conduct, including its resettlement policy in Mozambique. As awareness increased, Mozambican and Brazilian NGOs, with the support of international NGOs (e.g., Oxfam and ActionAid) and regional mining movements (e.g., the Southern African Alternative Mining Indaba), began coordinating action against Vale (Cezne, 2019; Milhorance & Bursztyn, 2017). Joint strategies included publishing human rights and environmental reports, shareholder activism through the purchase of Vale stocks, and field visits to Mozambique to provide eyewitness accounts. Mozambican civil society also provided legal support to communities impacted by Vale’s operations and connected with Danish and Canadian NGOs to pressure pension funds to divest from their Vale stocks.

AV’s resistance to Vale, however, slowed dramatically after 2015 as the political conditions in Brazil and Mozambique became increasingly hostile and unfavorable (Cezne, 2019). In Brazil, economic crisis, political instability, and the 2015 and 2019 Vale dam disasters diverted Brazilian civil society’s attention and resources to the domestic sphere. In Mozambique, meanwhile, the government since 2013 has increased surveillance and cracked down on dissent in response to increasing tension with the opposition party and insurgencies in the resource-rich Cabo Delgado province (Cezne, 2019; Lesutis, 2019a, 2019b).

Anti-ProSAVANA activism began around 2012 when Mozambican organizations became aware of the agreement signed two years earlier without the consent of affected communities. With the support of international NGOs, Mozambican groups connected with Brazilian NGOs through participation in AV, the Via Campesina food sovereignty movement, and Rio+20 People’s Summit. In the name of both international solidarity and a common struggle (because the project could trigger an exodus of Brazilian farmers and firms to Mozambique that would compromise food security in Brazil), Brazilian NGOs worked to unite relevant groups to fight against Brazil’s agribusiness activities in Mozambique (Aguiar & Pacheco, 2016).

Mozambique’s National Union of Peasants also publicly opposed the ProSAVANA project and, after learning that ProSAVANA was similar to the PROCEDER project, an Oxfam-funded civil society delegation from Mozambique and Brazil traveled to the Cerrado region of Brazil to survey the detrimental health and environmental impacts of PROCEDER. Upon returning to Mozambique, with a film crew documenting the trip, a domestic coalition authored an open letter demanding greater transparency, accountability, and respect for the autonomy of rural farmers (Aguiar & Pacheco, 2016; Durán & Chichava, 2017).

In 2014, Mozambican civil society groups launched the *No to ProSAVANA* campaign, which was promoted by their counterparts in Brazil and international NGOs. Parallel to these efforts, Mozambican civil society groups with a less critical stance against ProSAVANA used Brazil’s public commitment to fight hunger to lobby for social protections and consideration of family farming traditions within the project (Milhorance & Bursztyn, 2017). This plurality of tactics against the ProSAVANA project resulted in some increased transparency, including more consultations with affected farmers, and while it did not stop the project entirely, certain aspects of it were discontinued (Aguiar & Pacheco, 2016; Cezne, 2019).

## Conclusions

Our analysis provides a high-level overview of BRICS–South advocacy networks opposing BRICS extractive projects, including offering insights into the conditions under which these networks form and evolve. As one would expect, when the conditions for activism are more favorable in the South state than in the BRICS state, local groups in the South tend to mobilize and lead efforts to oppose BRICS extractive projects. That is what has occurred in the case of activism to oppose Chinese extractive projects in Ecuador. In this instance, the BRICS–South TAN is largely nonexistent.

These findings, we should note, stand in slight contrast to Yeophantong’s (2013), who reveals how nascent TANs in the Mekong region have managed to alter the course of some Chinese hydropower projects. As controversy swirled around the consequences of the Chinese-financed Myitsone hydropower dam in Myanmar, for instance, a network of civil society groups in Myanmar (e.g., Eco Dev) and regional NGOs (e.g., Salween Watch based in Chiang Mai, Thailand) began calling for the termination of the project. In solidarity with this network, Chinese civil society organizations managed to get the Chinese media to cover these anti-dam efforts (Yeophantong, 2013).

The decision in 2011 by then Myanmar President Thein Sein to suspend the dam surprised the developer, China Power Investment Corporation, and triggered debates in Chinese academic and policy circles about China’s responsibilities for overseas investments. The following year, the Chinese NGO Global Environmental Institute helped organize a meeting between a vice-minister of the Chinese Ministry of Environmental Protection and Myanmar’s Eco Dev NGO to discuss the issues surrounding the Myitsone dam. This example illustrates that China–South TANs can form and wield some influence (Yeophantong, 2013). Noting the absence of a China–Ecuador TAN versus a very early stage China–Myanmar TAN, future researchers may want to explore why, when, and under what conditions China–South TANs form, perhaps considering whether regional proximity may influence the viability of a China–South TAN.

Unlike in Ecuador, where activism to oppose Chinese extractive projects is primarily running through Ecuadorian channels, advocacy against Indian agricultural investments in Ethiopia have predominantly taken place outside of Ethiopia. In Ethiopia, repression of Indigenous peoples and limited human rights have almost entirely prohibited the multifaceted forms of resistance seen in Ecuador. Although repression of Indian civil society has been rising in recent years, some Indian NGOs are nevertheless continuing to work to expose the overseas impacts of India’s companies and development policies, especially in conflict settings. Because India’s outward-facing anti-extractive TAN is still in its early form, however, international organizations continue to play an important role in opposing Indian agricultural investments in Ethiopia. The India–Ethiopia case suggests that when the conditions are slightly better in the BRICS state, activism would seem more likely to take place at either the international level through North-based (including diaspora-led) NGOs or, to a growing degree, in the BRICS context. In this case, the BRICS–South TAN is strained, but emerging.

The breadth of Brazilian and Mozambican civil society strategies to resist Vale’s coal mines and the ProSAVANA project in Mozambique stands in contrast to the largely one-sided mobilizations that have taken place to resist China’s extraction in Ecuador and India’s extraction in Ethiopia. This Brazil–Mozambique case exemplifies that an active and strong BRICS–South TAN would seem to require reasonably favorable conditions in both domestic contexts. Under such conditions, members of the Brazil-Mozambique TANs have been able to develop dense, diverse, and durable ties as well as establish consistent channels of communication, which are all features of influential TANs (Keck & Sikkink, 1998, pp. 28–29; Shawki, 2011). Another facilitating feature is that the TANs were generally able to coalesce around a goal to end—or at least modify the trajectory of—the Vale and ProSAVANA projects. In line with our argument of the importance of favorable local conditions for empowering TANs, as civil society repression and political and economic instability have increased in Brazil and Mozambique since 2015, the frequency and intensity of the activities of TANs have declined.

For their part, NGOs based in the global North would seem to adjust their role depending on the particular strengths and gaps of the BRICS–South TANs. This may entail, among other activities, connecting global South NGOs in coalitions, researching and publishing reports, and facilitating and financing BRICS-based or South-based advocacy strategies. In addition, North-based organizations may be able to assist BRICS civil society organizations wanting to develop outward-facing advocacy networks, including connecting them with relevant South-based NGOs. Even without the assistance of North-based NGOs, however, a BRICS–South advocacy network can still mobilize against extractive projects when conditions in both civil societies favorably align.

In sum, our analysis highlights the crucial importance of local politics and the nature of civil society for shaping the formation, resiliency, and influence of BRICS–South advocacy networks that are opposing BRICS extractive projects in the global South. As the campaigns against BRICS extractive projects in Ecuador, Ethiopia, and Mozambique demonstrate, local characteristics shape the extent of cross-national cooperation, the role of North-based nonprofits, and the leadership positions of local groups within an advocacy network. Within each state, critical local characteristics include the degree of political repression and legal protections for NGOs, the extent of socioeconomic and political stability, the experience of NGOs and Indigenous groups in resisting large-scale corporate projects, and the value that local NGOs place on building South–South solidarity. These conclusions are not meant to close the book on this subject. The consequences of local conditions for transnational advocacy vary considerably, and our hope is future researchers will build on our findings to investigate additional factors that might be influencing the power and reach of BRICS–South advocacy networks, such as differing mobilization opportunities and constraints across extractive industries.

## Notes

1. Cooperation and solidarity between the BRICS and non-BRICS states of the global South are typically framed as South–South relations. As this article specifically explores the transnational advocacy networks between the BRICS and non-BRICS states in the global South, it refers to them separately, including through use of “BRICS–South” rather than “South–South.” We use this framing to communicate our argument and are not intending to convey that BRICS states are not part of the global South. We use the term global North, meanwhile, to refer to developed countries, but again recognize this delineation washes out significant differences.

2. That said, as others have found, South-based business and political elites do have considerable negotiating power to shape BRICS engagement and financial flows (Dye & Alencastro, 2020; Mohan & Lampert, 2013). Our thanks to an anonymous reviewer for this point.

3. See Naik (2018) and Telo (2017) for scholarship using this definition of BRICS.

4. Indicative of the increasingly repressive political settings for NGOs, according to Freedom House (2021), political rights, civil liberties, and democratic institutions have been steadily declining since 2006; by the beginning of 2021, less than 20% of the world’s population was in a “free” country, the lowest percentage since 1995.

5. Of course, adequate extraterritorial corporate legal obligations in the global North are rare too, even after decades of advocacy (Simons & Macklin, 2014).

6. Karuturi Global has a controversial history in Ethiopia. The company initially leased 300,000 ha of land in 2009, but this was reduced to 100,000 ha following an international media backlash. In 2017, the Ethiopian government canceled the land agreement with Karuturi Global because of under-cultivation. After diplomatic intervention by the Indian government and lawsuits filed by the company, however, in 2019, the Ethiopian government granted a new lease for 15,000 ha of land.
